# Plant hormone profiling of scion and rootstock incision sites and intra- and inter-family graft junctions in *Nicotiana benthamiana*

**DOI:** 10.1080/15592324.2024.2331358

**Published:** 2024-03-21

**Authors:** Kohei Kawaguchi, Michitaka Notaguchi, Koji Okayasu, Yu Sawai, Mikiko Kojima, Yumiko Takebayashi, Hitoshi Sakakibara, Shungo Otagaki, Shogo Matsumoto, Katsuhiro Shiratake

**Affiliations:** a Graduate School of Bioagricultural Sciences, Nagoya University, Nagoya, Japan; b RIKEN Center for Sustainable Resource Science, Plant Productivity Systems Research Group, Yokohama, Japan

**Keywords:** *Nicotiana benthamiana*, intrafamily grafting, interfamily grafting, hormonome analysis, auxin, cytokinin, gibberellic acid

## Abstract

Many previous studies have suggested that various plant hormones play essential roles in the grafting process. In this study, to understand the plant hormones that accumulate in the graft junctions, whether these are supplied from the scion or rootstock, and how these hormones play a role in the grafting process, we performed a hormonome analysis that accumulated in the incision site of the upper plants from the incision as “ungrafted scion” and lower plants from the incision as “ungrafted rootstock” in *Nicotiana benthamiana*. The results revealed that indole-3-acetic acid (IAA) and gibberellic acid (GA), which regulate cell division; abscisic acid (ABA) and jasmonic acid (JA), which regulate xylem formation; cytokinin (CK), which regulates callus formation, show different accumulation patterns in the incision sites of the ungrafted scion and rootstock. In addition, to try discussing the differences in the degree and speed of each event during the grafting process between intra- and inter-family grafting by determining the concentration and accumulation timing of plant hormones in the graft junctions, we performed hormonome analysis of graft junctions of intra-family grafted plants with *N. benthamiana* as scion and *Solanum lycopersicum* as rootstock (*Nb/Sl*) and inter-family grafted plants with *N. benthamiana* as scion and *Arabidopsis thaliana* as rootstock (*Nb/At*), using the ability of *Nicotiana* species to graft with many plant species. The results revealed that ABA and CK showed different accumulation timings; IAA, JA, and salicylic acid (SA) showed similar accumulation timings, while different accumulated concentrations in the graft junctions of *Nb/Sl* and *Nb/At*. This information is important for understanding the molecular mechanisms of plant hormones in the grafting process and the differences in molecular mechanisms between intra- and inter-family grafting.

## Introduction

Grafting is a common horticultural technique in which tissues from two individual plants are connected to form a new chimeric plant.^1^ Grafting provides benefits, such as improving nutrient uptake, resistance to diseases and pests, plant growth, and fruit quality.^1–4^ Grafting is widely used in the commercial production of *Solanaceae* crops, such as tomato (*Solanum lycopersicum*), eggplant (*Solanum melongena*), and chili pepper (*Capsicum annuum*), *Cucurbitaceae* crops, such as watermelon (*Citrullus lanatus*), melon (*Cucumis melo*), and cucumber (*Cucumis sativus*), as well as the vegetative propagation of fruit trees, including apple (*Malus domestica*), citrus *(Citrus sinensis)*, grape (*Vitis vinifera*), mango (*Mangifera indica*), apricot (*Prunus armeniaca*), peache (*Prunus persica*), pear (*Pyrus sativa*), persimmon (*Diospyros kaki*), plum (*Elaeis guineensis*), sweet cherry (*Cerasus avium*), and walnut (*Juglans hindsii*).^5–7^ In recent years, grafting has also been used in plant research as a tool to study the systemic signaling and interorgan transport of diverse endogenous molecules, including phytohormones, metabolites, small RNAs, messenger RNAs, secreted peptides, and proteins.^8–10^ Grafting is established through the following processes in the graft junction between the scion and rootstock: formation and degradation of the necrotic layer owing to collapsed cells, cell division and callus formation, intercellular junctions, plasmodesmata connection, and reformation and reconnection of vascular bundles.^11,12^ Using functional assay in the grafting junction of *Arabidopsis thaliana* that measures the movement of fluorescent molecules, the phloem reconnected 3–4 days after grafting (DAG), and the xylem reconnected 6–8 DAG, suggesting this process was completed about one week after grafting.^13^ These grafting processes are similar to the tissue reunion processes of the incised stem.^14^

Various plant hormones play essential roles in tissue reunion and grafting.^14^ Auxin regulates cell division and vascular bundle formation in tissue reunion of the incised stem and grafting.^15–17^ In incised Arabidopsis inflorescence stems, auxin transport from the apical bud to the root is blocked, and auxin levels increase in the upper part from the incised, whereas the auxin level decreases in the lower part from the incised.^15^ In the upper part from incised, auxin increases the expression of *AUXIN RESPONSE FACTOR* (*ARF*) *6* and *ARF8*, promoting the expression of *ARABIDOPSIS NAC DOMAIN CONTAINING PROTEIN* (*ANAC*) *071*.^15^ In addition, ANAC071 binds directly to the promoter sites of *XYLOGLUCAN ENDO-TRANSGLUCOSYLASE/HYDROLASE* (*XTH*) *19* and *XTH20* and increases their expression, resulting in the promotion of cell division of pith tissue during the tissue reunion process in incised inflorescence stems.^15,18^ The auxin accumulation pattern in the upper and lower parts of incised Arabidopsis inflorescence stems was also observed in the graft junctions of the Arabidopsis hypocotyl based on the gene expression pattern of auxin-responsive transcription factors.^17^ Furthermore, ANAC071 and its homolog ANAC096 redundantly regulate cell division in vascular tissues during Arabidopsis hypocotyl grafting,^17^ indicating that the grafting processes are similar to the tissue reunion processes of the incised stem.

Cytokinin (CK) plays an essential role in callus and vascular bundle formation. In the wound site of Arabidopsis hypocotyls, CK levels increase due to the promotion of CK biosynthesis gene expression.^19,20^ Furthermore, WOUND-INDUCED DEDIFFERENTIATION 1 (WIND1), a wound-induced AP2/ERF transcription factor, activates CK signaling and promotes wound-induced callus formation.^19,20^ In addition, CK-deficient mutants exhibit reduced callus formation.^20^ These results indicate that CK plays an essential role in callus formation via the CK signaling pathway in the wound response. CK also regulates cell division in a dose-dependent vascular cambium.^21^ Decreasing the CK level inhibits cambium cell division and induces thinner stems,^22^ whereas increasing the CK level promotes CK signaling and cambium cell division activity in poplar.^23^ In addition, the Arabidopsis CK receptor *CYTOKININ RESPONSE 1* (*CRE1*)*/WOODEN LEG* (*WOL*)*/ARABIDOPSIS HISTIDINE KINASE* (*AHK*) *4* mutant or *AHK2*, *AHK3*, and *AHK4* triple mutants decreased cambial cell division activity in the root vascular bundle.^24,25^ CK interacts with the auxin signaling pathway.^26–28^ CK regulates auxin transport due to the distribution of auxin transporter PIN proteins in vascular bundle formation in developing Arabidopsis vascular tissue.^29^ Auxin promotes xylem formation by increasing the expression of the CK signaling inhibitor *ARABIDOPSIS HISTIDINE PHOSPHOTRANSFER PROTEIN 6* (*AHP6*).^30^ Several CK signaling mutants induced extra xylem, whereas exogenous CK treatment reduced xylem formation,^31,32^ suggesting that CK interacts antagonistically with auxin to regulate xylem formation negatively. These previous studies suggest that various plant hormones, including auxin and CK, play an essential role in the grafting process through callus formation, cell division, and vascular bundle formation.

Grafting is most successful between plants of the same family.^11,33^ However, *Nicotiana* species exhibited compatibility in inter-family grafting with 73 species from 38 families, including important vegetables, flowers, and fruit tree crops.^12^ Transcriptome analysis of graft junctions in various grafted plants with *Nicotiana benthamiana* revealed that *GH9B3* encoding β-1,4-glucanase secreted to the extracellular region was highly expressed in the graft junction of inter-family grafted plants with *N. benthamiana* as scion and *A. thaliana* as rootstock (*Nb/At*) than *N. benthamiana* self-grafted plants (grafted with the same plant as the scion and rootstock).^12^ β-1,4-glucanase promoted self- and inter-family grafting through necrotic layer degradation and cell wall reconstruction in the graft junctions.^12^ In a previous study by Notaguchi et al.,^12^ the gene expression of the auxin-responsive transcription factors IAA1 and ANAC071 increased in the *Nb/At* compared with the *N. benthamiana* self-grafted plants in the graft junctions. These results suggest that the accumulation of plant hormones, such as auxin, may differ depending on the degree and speed of events during the grafting process.

As described above, many previous studies have suggested that various plant hormones play an essential role in grafting; however, no comprehensive analysis of plant hormones accumulated in the graft junction, including in the model plant *A. thaliana*, has been reported. Therefore, we performed hormonome analysis in the incision sites of the ungrafted scion and rootstock in *N. benthamiana* to understand the plant hormones that accumulate in the graft junctions, whether these are supplied from the scion or rootstock, and how these hormones play a role in the grafting process. In addition, we performed hormonome analysis of the graft junctions of intra-family grafting (*Nb/Sl*: grafted plants with *N. benthamiana* as scion and *S. lycopersicum* as rootstock) and inter-family grafting (*Nb/At*: grafted plants with *N. benthamiana* as scion and *A. thaliana* as rootstock) to try discussing the differences in the degree and speed of each event during the grafting process between intra- and inter-family grafting by determining the concentration and accumulation timing of plant hormones in the graft junctions.

## Materials and methods

### Plant materials and growth conditions

*Nicotiana benthamiana*, *Arabidopsis thaliana* Col-0, and *Solanum lycopersicum* ‘Moneymaker’ were used for the experiments. *N. benthamiana* and *A. thaliana* seeds were surface sterilized with 5% (w/v) bleach for 5 min, washed three times with sterile water, incubated at 4°C for three days, and sown on 1/2 Murashige and Skoog medium (pH 5.8) contains 0.5% (w/v) sucrose and 1% (w/v) agar. These seedlings were grown on the medium for one week and transplanted into the soil. *S. lycopersicum* seeds were directly sown on the soil. *A. thaliana* seedlings were grown at 22°C, and *S. lycopersicum* and *N. benthamiana* seedlings were grown at 27°C under continuous light of 100 μmol/m^2^/s in the growth chamber.

### Sampling of incision sites of ungrafted scion and rootstock

All procedures were the same as grafting except for the scion and rootstock tissue adhesion. Four to five-week-old *N. benthamiana* inflorescence stem was incision horizontally in the middle using a razor blade, and leaves near the incision site were removed. The upper plant from the incision site was used as a ungrafted scion, and the lower plant from the incision site was used as a ungrafted rootstock The ungrafted scion was incision into a V-shape and wrapped with parafilm at the incision site of the inflorescence stem, supported by a plastic pole to keep it vertical, sprayed water, and then covered with a plastic bag to keep humidity. The ungrafted rootstock was incision to make a 2 cm slit at the incision site of the inflorescence stem and covered with a plastic back. The incision site of the ungrafted rootstock was not wrapped with parafilm to avoid self-tissue adhesion of the slit. The ungrafted scion and rootstock were grown at 27°C under continuous light (−30 µmol/m^2^/s) in the growth chamber. The incision sites of the ungrafted scion and rootstock were sampled 2, 24, 72, 120, or 168 hours after cutting (HAC) with the intact stem as the control. Four biological replicates of each sample were analyzed.

### Sampling of graft junction

Four- to five-week-old *N. benthamiana* inflorescence stems, five-week-old *A. thaliana* inflorescence stems, and four-week-old *S. lycopersicum* stems were used. Grafting and sampling were performed according to Notaguchi et al.. (2020), and intra-family grafted plants with *N. benthamiana* as scion and *S. lycopersicum* as rootstock (*Nb/Sl*) and inter-family grafted plants with *N. benthamiana* as scion and *A. thaliana* as rootstock (*Nb/At*) were performed by splice grafting. The graft junctions of *Nb/Sl* and *Nb/At* were sampled 2, 24, 72, 120, or 168 hours after grafting (HAG) with the intact stem of *N. benthamiana* as control. Four biological replicates of each sample were analyzed.

### Plant hormonome analysis

The incision sites of ungrafted scion and rootstock and graft junctions of *Nb/Sl* and *Nb/At* were grounded in liquid nitrogen and freeze-dried and then analyzed for plant hormonome analysis. *Nb/At* graft junction samples were also used for the transcriptomics by Notaguchi et al..^12^ Plant hormone extraction and fractionation were performed according to Kojima et al.^34^ and Kojima and Sakakibara.^35^ CKs were quantified by the ultra-performance liquid chromatography-electrospray interface and tandem quadrupole mass spectrometer (UPLC-ESI-qMS/MS) (AQUITY UPLC System/Xevo-TQS, Waters, Massachusett, USA) as described by Kojima et al..^34^ Abscisic acid (ABA), indole-3-acetic acid (IAA), indole-3-acetyl aspartate (IAAsp), jasmonic acid (JA), salicylic acid (SA), and gibberellic acid (GA) were quantified by the ultra-high performance-liquid chromatography electrospray interface and quadrupole-orbitrap mass spectrometer (UHPLC-ESI-Q-Orbitrap-MS/MS) (UltiMate 3000/Q-Exactive, Thermo Scientific, Massachusetts, USA) as described by Kojima and Sakakibara^35^ and Shinozaki et al..^36^

### Statistical analysis

Multiple tests were performed by R statistical software version 4.2.1 (R Project for Statistical Computing, https://www.r-project.org/) within RStudio statistical software version 2022.2.3.492 (RStudio, https://www.rstudio.com/) using two-tailed Tukey-Kramer test and *p* ≤0.05 was considered as a significant difference.

A two-tailed Student’s *t*-test was performed by Microsoft EXCEL 2019, and *p* ≤ 0.05 was considered a significant difference.

## Results and discussion

### Plant hormonome analysis at incision sites of the scion and rootstock

To understand the accumulation of plant hormones at graft junctions, whether hormones are supplied from the scion or rootstock, and the role of hormones in the grafting process, we performed hormonome analysis of accumulation at the incision sites of the scion and rootstock in *N. benthamiana*. During the grafting process, the tissue at the graft junction adheres and the vascular bundles of the scion and rootstock reconnect, transferring plant hormones between the scion and rootstock. Thus, whether plant hormones are supplied to the graft junction from the scion or the rootstock is unclear.

In the present study, we comprehensively analyzed plant hormones that accumulated at the incision site in the plant parts above the incision considered the “ungrafted scion” and plant parts below the incision considered the “ungrafted rootstock” prepared by incision *N. benthamiana* inflorescence stems. The results revealed that SA and GA_1_ were not detected at the incision sites of the ungrafted scion or rootstock, whereas auxins (IAA and IAAsp), CKs, ABA, GA_4_, and JA showed different accumulation patterns at the incision sites of the ungrafted scion and rootstock. The details and discussion are presented in the following sections.

### Auxins

#### Indole-3-acetic acid accumulates at the incision site of the scion

**Figure 1.**
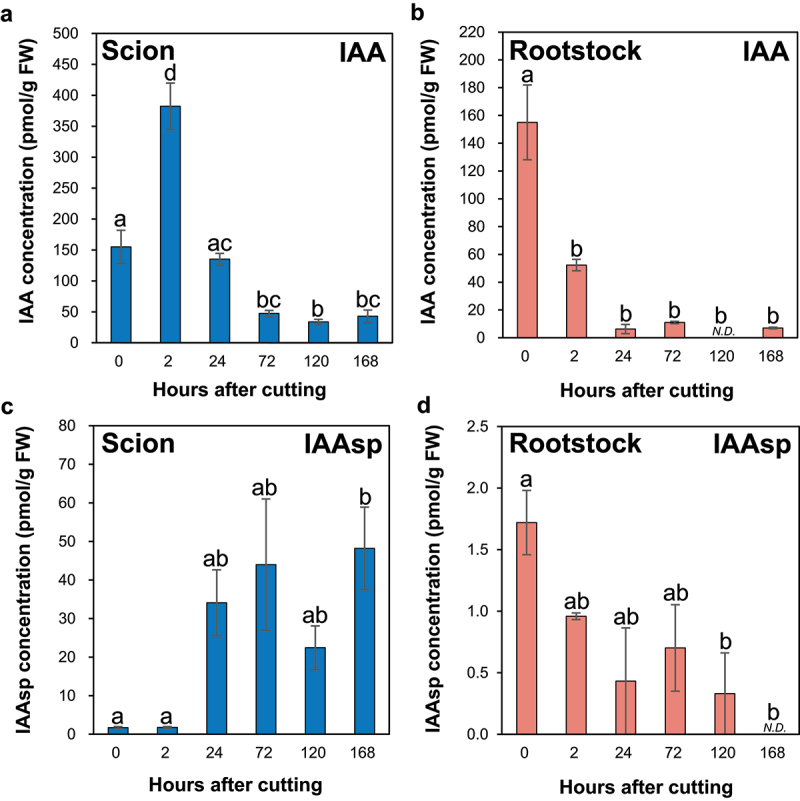
The auxin concentrations at the incision sites of the ungrafted scion and rootstock of *N. benthamiana*. The concentrations of the IAA (a,c) and IAAsp (b,d) at the incision sites of the ungrafted scion or rootstock. Blue and pink indicate ungrafted scion and rootstock, respectively. Different letters indicate significant differences according to the Tukey-Kramer test (*p* ≤ 0.05). Values are the means of four biological replicate samples, and error bars indicate the standard error of four biological replicate samples. *N.D*. indicates not detected.

The IAA concentration significantly increased by approximately 2.5-fold at 2 h after cutting (HAC) at the incision site of the ungrafted scion compared with the control (Figure 1(a)), whereas the IAA concentrations significantly decreased to 1/3 at 2 HAC at the incision site of the ungrafted rootstock compared with the control (Figure 1(b)).

In incised Arabidopsis inflorescence stems, auxin transport from the apical bud to the root was blocked, and auxin levels increased in the part above the incision, whereas auxin levels decrease in the lower part.^15^ In the part above the incision, auxin increased the expression of *ARF6* and *ARF8*, thereby promoting the expression of *ANAC071*.^15^ In addition, ANAC071 binds directly to the promoter sites of *XTH19* and *XTH20* and increases their expression, promoting cell division in the pith tissue during the tissue reunion process in incised inflorescence stems.^15,18^ The auxin accumulation pattern in the upper and lower parts of incised Arabidopsis inflorescence stems was also observed in the graft junctions of the Arabidopsis hypocotyl, based on the gene expression pattern of auxin-responsive transcription factors.^17^ Furthermore, ANAC071 and its homolog ANAC096 redundantly regulate cell division in vascular tissues during the Arabidopsis hypocotyl grafting processes,^17^ indicating that auxin plays an essential role in the tissue reunion and grafting processes of incised stems. The IAA accumulation pattern at the incision sites of the ungrafted scion and rootstock was similar to that in the upper and lower parts of Arabidopsis inflorescence stems and graft junctions in Arabidopsis hypocotyls (Figure 1(a,b)).

In addition, a previous study by Notaguchi et al.^12^ observed that the gene expression of the auxin-responsive transcription factors IAA1 and ANAC071 increased in the graft junction of self-grafted *N. benthamiana* plants at 1 DAG. These results suggest that the grafting process through auxin accumulation and auxin-responsive transcription factors was present in both *A. thaliana* and *N. benthamiana.*

#### Indole-3-acetic acid was inactivated by metabolizing to IAAsp at the incision site of the scion

The IAAsp concentration at the incision site of the ungrafted scion tended to increase at 24, 72, and 120 HAC, and significantly increased at 168 HAC compared to the control (Figure 1(c)). In contrast, the IAAsp concentration at the incision site of the ungrafted rootstock significantly decreased at 24, 120, and 168 HAC compared to that of the control (Figure 1(d)).

The family of acyl acid amido synthetases GH3 catalyzes the conversion of IAA to IAA-amino acid conjugates such as IAAsp and indole-3-acetyl glutamate and plays a central role in IAA inactivation.^37^ Compared to the control, the concentration of IAA at the incision site of the ungrafted scion significantly increased at 2 HAC, significantly decreased at 72, 120, and 168 HAC, and the change was not significant at 24 HAC (Figure 1(a)), suggesting that IAA accumulated at 2 HAC, whereas it was metabolized to IAAsp by GH3 at 2 HAC onward at the incision site of the scion. Therefore, IAA accumulation and IAA inactivation of IAAsp by GH3 at the incision site of the scion may be involved in the grafting process.

### Cytokinins

#### Cks, iP- and tZ-type, accumulate at the incision site of the rootstock

Cytokinins can be classified into four types: N6-(Δ 2-isopentenyl)-adenine (iP), *trans*-zeatin (tZ), *cis*-zeatin (cZ), and dihydrozeatin (DZ) types. The active CKs are iP, tZ, and DZ, whereas as a bioactive CK, cZ is expected to be significantly low or absent.^38^ The total concentration of the cZ- and DZ-type CKs at the incision sites of the ungrafted scion and rootstock at any time point was less than 7 pmol/g fresh weight (FW) and 4 pmol/g FW, respectively, lower than that of the total concentration of the iP- and tZ-type CKs, and active CK DZ was not detected in most samples (Tables 1 and 2). These results suggest that cZ- and DZ-type CKs may not be involved in the grafting process.

**Table 1.** The CK concentrations in the incision site of the ungrafted scion of *N.benthamiana*.

	Control	Scion_2 h	Scion_24 h	Scion_72 h	Scion_120 h	Scion_168 h
iP	*N.D*. a	*N.D*. a	*N.D*. a	0.11 ± 0.11 a	*N.D*. a	*N.D*. a
iPR	0.28 ± 0.05 a	0.32 ± 0.02 a	0.14 ± 0.05 a	0.92 ± 0.07 a	5.45 ± 0.48 b	8.50 ± 0.88 c
iPRPs	17.57 ± 4.23 ab	13.72 ± 0.86 bd	4.02 ± 1.38 d	6.31 ± 0.80 d	26.30 ± 1.85 ac	34.63 ± 2.94 c
iP7G	3.17 ± 0.32 a	4.61 ± 0.48 a	7.74 ± 1.56 a	13.38 ± 1.62 a	29.01 ± 3.99 b	39.26 ± 4.96 b
iP9G	*N.D*. a	*N.D*. a	*N.D*. a	*N.D*. a	*N.D*. a	*N.D*. a
tZ	0.46 ± 0.09 a	0.43 ± 0.02 a	0.13 ± 0.06 a	0.18 ± 0.18 a	0.11 ± 0.02 a	0.13 ± 0.02 a
tZR	0.87 ± 0.12 a	1.17 ± 0.12 bd	0.07 ± 0.05 c	*N.D*. c	0.60 ± 0.06 a	0.79 ± 0.09 ab
tZRPs	10.40 ± 2.15 a	12.30 ± 1.14 a	0.46 ± 0.17 b	0.22 ± 0.02 b	1.38 ± 0.12 b	1.89 ± 0.12 b
tZ7G	0.92 ± 0.16 a	1.29 ± 0.16 ab	1.60 ± 0.34 ac	2.08 ± 0.11 bc	1.51 ± 0.05 ab	2.59 ± 0.29 c
tZ9G	*N.D*. a	*N.D*. a	*N.D*. a	*N.D*. a	*N.D*. a	*N.D*. a
tZOG	0.06 ± 0.01 a	0.05 ± 0.01 ac	0.01 ± 0.01 bc	*N.D*. b	*N.D*. b	*N.D*. b
tZROG	0.13 ± 0.03 a	0.13 ± 0.01 a	0.15 ± 0.02 a	0.18 ± 0.02 a	0.15 ± 0.02 a	0.17 ± 0.02 a
tZRPsOG	*N.D*. a	*N.D*. a	*N.D*. a	*N.D*. a	*N.D*. a	*N.D*. a
cZ	0.48 ± 0.37 a	0.05 ± 0.01 a	0.09 ± 0.02 a	0.17 ± 0.02 a	0.12 ± 0.04 a	0.10 ± 0.05 a
cZR	0.10 ± 0.02 a	0.51 ± 0.09 a	0.37 ± 0.05 a	0.76 ± 0.05 b	0.84 ± 0.05 b	0.87 ± 0.02 b
cZRPs	0.97 ± 0.12 a	1.94 ± 0.32 ac	1.37 ± 0.21 ab	1.84 ± 0.12 ac	2.23 ± 0.20 bc	2.41 ± 0.25 c
cZOG	0.01 ± 0.01 a	0.03 ± 0.01 a	0.05 ± 0.05 a	0.04 ± 0.04 a	0.08 ± 0.05 a	0.10 ± 0.08 a
cZROG	0.14 ± 0.03 a	0.14 ± 0.03 a	0.18 ± 0.01 a	0.22 ± 0.02 ab	0.19 ± 0.02 a	0.32 ± 0.03 b
cZRPsOG	*N.D*. a	0.06 ± 0.00 ac	0.12 ± 0.01 ac	0.25 ± 0.02 ac	0.34 ± 0.02 bc	0.61 ± 0.17 b
DZ	*N.D*. a	*N.D*. a	0.01 ± 0.01 a	*N.D*. a	*N.D*. a	*N.D*. a
DZR	0.01 ± 0.01 a	*N.D*. a	*N.D*. a	*N.D*. a	*N.D*. a	*N.D*. a
DZRPs	0.13 ± 0.03 a	0.13 ± 0.02 a	0.06 ± 0.03 ab	*N.D*. b	*N.D*. b	*N.D*. b
DZ9G	0.08 ± 0.02 a	0.03 ± 0.02 ab	0.07 ± 0.03 ab	0.10 ± 0.01 ab	0.07 ± 0.01 b	0.06 ± 0.03 ab

The concentrations of iP, iPR, iPRs, iP7G, iP9G, tZ, tZR, tZRPs, tZ7G, tZ9G, tZOG, tZROG, cZ, cZR, cZOG, cZROG, DZ, DZR, DZRPs, and DZ9G in the incision site of the ungrafted scion. Different letters indicate significant differences according to the Tukey-Kramer test (*p* ≤ 0.05). Values are the means of four replicate samples. All concentrations are means and ± indicates the standard error of four replicate samples. *N.D*. indicates not detected.

**Table 2.** The CKs concentration in the incision site of the ungrafted rootstock of *N. benthamiana.*

	Control	Stock_2 h	Stock_24 h	Stock_72 h	Stock_120 h	Stock_2 h
iP	*N.D*. a	*N.D*. a	0.93 ± 0.10 c	0.49 ± 0.06 b	0.33 ± 0.01 b	0.30 ± 0.02 b
iPR	0.28 ± 0.05 a	0.25 ± 0.03 a	20.82 ± 6.63 b	13.51 ± 3.23 ab	3.48 ± 0.40 a	5.65 ± 1.66 a
iPRPs	17.57 ± 4.23 a	12.87 ± 2.42 a	374.47 ± 58.16 c	169.34 ± 23.93 b	146.46 ± 1.56 b	103.14 ± 3.67 ab
iP7G	3.17 ± 0.32 a	4.44 ± 0.28 a	19.46 ± 0.78 ac	52.12 ± 10.84 bc	65.09 ± 3.41 b	83.95 ± 13.48 b
iP9G	*N.D*. a	*N.D*. a	*N.D*. a	*N.D*. a	*N.D*. a	*N.D*. a
tZ	0.46 ± 0.09 a	0.29 ± 0.03 a	0.35 ± 0.06 a	0.57 ± 0.13 a	0.29 ± 0.03 a	0.24 ± 0.07 a
tZR	0.87 ± 0.12 a	1.03 ± 0.25 a	5.77 ± 0.94 b	5.26 ± 0.84 b	2.19 ± 0.18 a	1.72 ± 0.49 a
tZRPs	10.40 ± 2.15 a	7.75 ± 0.86 a	36.66 ± 2.65 c	32.89 ± 6.80 bc	19.06 ± 2.07 ab	12.40 ± 2.79 a
tZ7G	0.92 ± 0.16 a	1.26 ± 0.05 a	1.65 ± 0.08 ab	3.41 ± 0.70 b	3.33 ± 0.13 b	3.58 ± 0.73 b
tZ9G	*N.D*. a	*N.D*. a	*N.D*. a	*N.D*. a	*N.D*. a	*N.D*. a
tZOG	0.06 ± 0.01 a	*N.D*. b	*N.D*. b	*N.D*. b	*N.D*. b	*N.D*. b
tZROG	0.11 ± 0.02 a	0.11 ± 0.02 a	0.15 ± 0.02 abc	0.56 ± 0.09 bd	0.59 ± 0.07 cd	0.71 ± 0.20 d
tZRPsOG	*N.D*. a	*N.D*. a	*N.D*. a	0.14 ± 0.02 b	0.22 ± 0.03 b	0.17 ± 0.04 b
cZ	0.48 ± 0.37 a	0.04 ± 0.01 a	0.04 ± 0.02 a	0.07 ± 0.01 a	0.02 ± 0.02 a	*N.D*. a
cZR	0.10 ± 0.02 a	0.35 ± 0.07 ab	0.88 ± 0.17 c	0.48 ± 0.06 b	0.16 ± 0.00 ab	0.11 ± 0.01 ab
cZRPs	0.97 ± 0.12 a	1.30 ± 0.20 ac	5.66 ± 0.29 d	4.14 ± 0.72 bd	2.84 ± 0.15 bc	1.69 ± 0.08 ac
cZOG	0.01 ± 0.01 a	*N.D*. a	*N.D*. a	*N.D*. a	*N.D*. a	*N.D*. a
cZROG	0.14 ± 0.03 a	0.11 ± 0.01 b	0.13 ± 0.01 b	0.30 ± 0.05 ac	0.42 ± 0.04 c	0.47 ± 0.05 c
cZRPsOG	*N.D*. a	0.02 ± 0.02 ad	0.10 ± 0.00 de	0.16 ± 0.02 e	0.38 ± 0.04 b	0.28 ± 0.01 c
DZ	*N.D*. a	*N.D*. a	*N.D*. a	*N.D*. a	*N.D*. a	*N.D*. a
DZR	0.01 ± 0.01 a	0.02 ± 0.01 a	0.16 ± 0.04 a	0.65 ± 0.16 b	0.32 ± 0.02 ab	0.22 ± 0.06 a
DZRPs	0.13 ± 0.03 a	0.09 ± 0.00 a	0.67 ± 0.16 a	2.31 ± 0.64 b	1.36 ± 0.20 ab	0.71 ± 0.16 a
DZ9G	0.08 ± 0.02 a	0.05 ± 0.00 ab	0.04 ± 0.02 ab	0.05 ± 0.01 ab	*N.D*. b	0.02 ± 0.02 ab

The concentrations of iP, iPR, iPRs, iP7G, iP9G, tZ, tZR, tZRPs, tZ7G, tZ9G, tZOG, tZROG, cZ, cZR, cZOG, cZROG, DZ, DZR, DZRPs, and DZ9G in the incision site of the ungrafted rootstock. Different letters indicate significant differences according to the Tukey-Kramer test (*p* ≤ 0.05). Values are the means of four replicate samples. All concentrations are means and ± indicates the standard error of four replicate samples. *N.D*. indicates not detected

**Figure 2.**
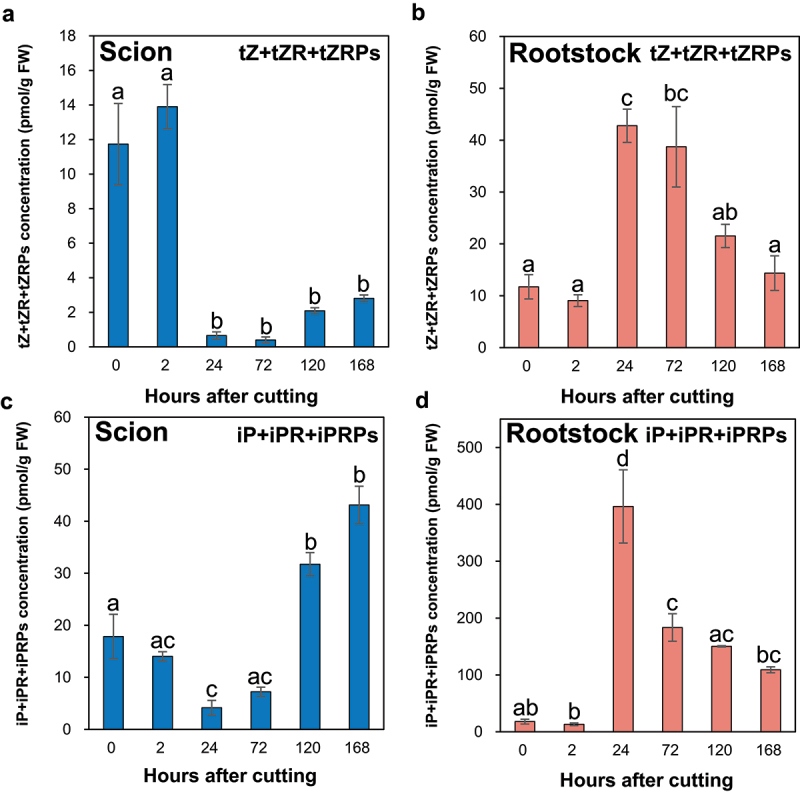
The major tZ- and iP-type CK concentrations at the incision sites of the ungrafted scion and rootstock of *N.benthamiana*. The total concentrations of the major tZ-type CKs (tZ, tZR, and tZRPs; a,c) and iP-type CKs (iP, iPR, and iPRPs; b,d) in the incision sites of the ungrafted scion and rootstock. Blue and pink indicate ungrafted scion and rootstock, respectively. Different letters indicate significant differences according to the Tukey-Kramer test (*p* ≤ 0.05). Values are the means of four biological replicate samples, and error bars indicate the standard error of four biological replicate samples.

Regarding tZ-type CKs, at the incision site of the ungrafted scion, tZ9G and tZRPsOG were not detected, tZOG and tZROG concentrations were less than 0.2 pmol/g FW, and tZ7G concentration increased over time from the time of incision (Table 1). The total concentrations of the major tZ-type CKs (tZ, tZR, and tZRPs) significantly decreased at 24, 72, 120, and 168 HAC compared to the control (Figure 2(a)). In contrast, at the incision site of the ungrafted rootstock, tZ9G and tZOG were not detected, tZRPsOG concentration was less than 0.2 pmol/g FW at any given time point, and tZ7G concentration increased over time from the time of incision (Table 2). The total concentrations of the major tZ-type CKs (tZ, tZR, and tZRPs) significantly increased by approximately 4-fold at 24 HAC compared to the control, significantly decreased at 120 and 168 HAC from 24 HAC and reached the same level as the control (Figure 2(b)). Regarding iP-type CKs, at the incision site of the ungrafted scion, iP and iP9G were not detected, and iP7G concentration significantly increased at 120 and 168 HAC compared to the control (Table 1). The total concentrations of the major iP-type CKs (iP, iPR, and iPRPs) significantly decreased by approximately 1/4 at 24 HAC compared with the control, and significantly increased from the level at 72 HAC until 120 and 168 HAC (Figure 2(c)). In contrast, at the incision sites of the ungrafted rootstock, the iP7G concentration increased from 72 to 168 HAC (Table 2), and the total concentrations of the major CKs (iP, iPR, and iPRPs) significantly increased by approximately 30-fold from 2 to 24 HAC and significantly decreased from 24 to 72 HAC (Figure 2(d)).

At the wound site of Arabidopsis hypocotyls, the wound-induced AP2/ERF transcription factor WIND1 activates CK signaling and tZ biosynthesis and promotes wound-induced callus and xylem formation.^19,20,39^ A homolog of WIND1 has been found in many plant species, suggesting that wound-induced callus and xylem formation through CK by WIND1 is preserved in many plant species.^40^ In contrast, iP promotes polar auxin transport and vascular bundle formation in root meristematic tissues and regulates root development.^29^

In the present study, iP- and tZ-type CK levels were increased at the incision site of the ungrafted rootstock compared with the control, consistent with results of previous studies by Iwase et al.^19^ and Ikeuchi et al.,^20^ which showed that stem wounding promoted the expression of CK synthesis genes and increased CK levels. Therefore, the increase in CK levels at the incision site of the ungrafted rootstock may play a crucial role in the grafting process through wound-induced callus and vascular bundle formation, including xylem formation.

#### Cytokinin and auxin accumulation presented a negative correlation at the incision sites of scion and rootstock

The IAA concentration significantly increased by approximately 2.5-fold at 2 HAC (Figure 1(a)), and the total concentrations of major iP- and tZ-type CKs significantly decreased at 24 HAC compared to the control at the incision site of the ungrafted scion (Figure 2(a,c)). In contrast, the IAA concentration significantly decreased by 1/3 at 2 HAC (Figure 1(b)), and the total concentrations of major iP- and tZ-type CKs significantly increased at 24 HAC compared to the control at the incision site of the ungrafted rootstock (Figure 2(b,d)). These results indicate a negative correlation between the concentrations of IAA and major iP- and tZ-type CK at the incision sites of the ungrafted scion and rootstock.

Cytokinin antagonistically interacts with the auxin signaling pathway.^26–28^ In addition, CK promotes callus and vascular bundle formation in the presence of auxins.^29^ In the present study, a negative correlation was observed between IAA and major iP- and tZ-type CK concentrations at the incision site of the ungrafted scion and rootstock, consistent with results of previous studies by Osugi and Sakakibara,^26^ Schaller et al.,^27^ and Kieber and Schaller,^28^ which showed that CK interacts antagonistically with the auxin signaling pathway. Therefore, CKs may interact antagonistically with auxin at the incision sites of the scion and rootstock and play an important role in the grafting process through callus and vascular bundle formation.

### Abscisic acid

#### Abscisic acid accumulates at the incision sites of the scion and rootstock

**Figure 3.**
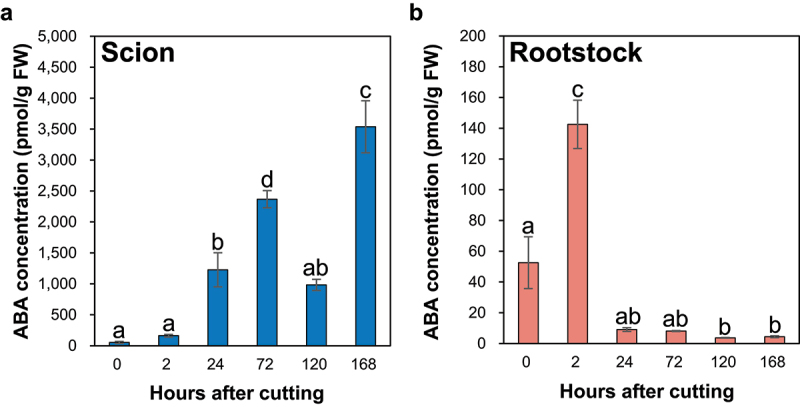
The ABA concentration at the incision sites of the ungrafted scion and rootstock of *N.benthamiana*. Blue and pink indicate ungrafted scion and rootstock, respectively. Different letters indicate significant differences according to the Tukey-Kramer test (*p *≤ 0.05). Values are the means of four biological replicate samples, and error bars indicate the standard error of four biological replicate samples.

The ABA concentration at the incision site of the ungrafted scion tended to increase over time and significantly increased at 24, 72, and 168 HAC compared with the control (Figure 3(a)). In contrast, compared with the control, the ABA concentration at the incision site of the ungrafted rootstock significantly increased by 2.7-fold at 2 HAC and significantly decreased from 2 HAC (Figure 3(b)).

The ABA concentration in the xylem sap increases under drought stress.^41^ In addition, the ABA concentration increases in the xylem and phloem sap under salt stress, and the ABA concentration is higher in the phloem sap than in the xylem sap.^42^ Most of the ABA accumulated in the roots is synthesized in the leaves,^43^ suggesting that ABA is synthesized primarily in leaf vascular cells, transported to the roots through the phloem, and further transported to the shoots through the xylem.^44^ In the present study, ABA concentrations at the incision site of the ungrafted scion were higher than those in the control, suggesting that the absence of roots in the ungrafted scion may induce drought stress owing to the loss of water supply to the plant from the root, leading to increased ABA synthesis in leaf vascular cells and transport through the phloem to the incision site of the ungrafted scion. Furthermore, ABA increases the expression of *miRNA165* and decreases the gene expression of class III homeodomain leucine zipper (HD-ZIP III) transcription factors, such as PHABULOSA (PHB) and *ARABIDOPSIS THALIANA* HOMEOBOX 8 (ATHB8), which promote xylem formation in the roots of *A. thaliana*.^45^ Therefore, ABA accumulation at the incision sites of scion and rootstock may play a crucial role in the grafting process via xylem formation.

### Gibberellic acid

#### Gibberellic acid accumulated at the incision site of the scion

**Figure 4.**
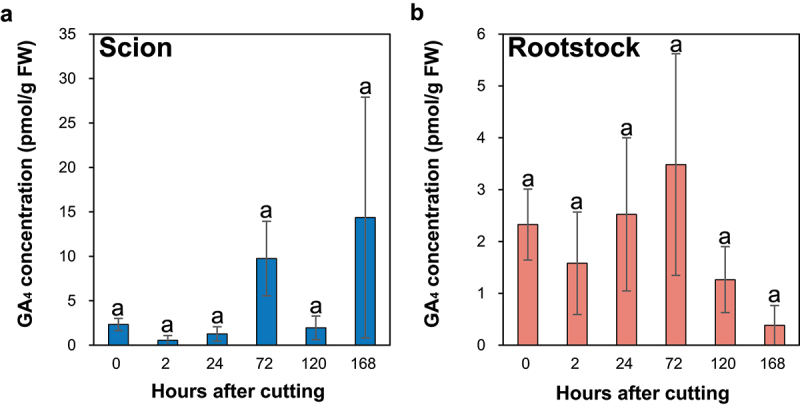
The GA₄ concentration at the incision sites of the ungrafted scion and rootstock of *N.benthamiana*. Blue and pink indicate ungrafted scion and rootstock, respectively. Different letters indicate significant differences according to the Tukey-Kramer test (*p *≤ 0.05). Values are the means of four biological replicate samples, and error bars indicate the standard error of four biological replicate samples.

GA_1_ was not detected at the incision sites of the ungrafted scions and rootstocks. The concentration of GA_4_ was not significantly different (Figure 4(a)); however, a tendency to increase at 72 HAC compared to the control at the incision site of the ungrafted scion (Figure 4(a)) and a tendency to decrease at 168 HAC compared to the control at the incision site of the ungrafted rootstock (Figure 4(b)) was observed.

In cucumber seedlings wounded below the cotyledons, removing the cotyledons strongly suppressed tissue reunion, whereas GA treatment of the apical bud induced tissue reunion at the wound site.^46^ This previous study suggested that GA synthesized in the cotyledons and transported to the wound site via the phloem promoted tissue reunion. In addition, treatment of cotyledons with the GA inhibitor, uniconazole, suppressed cortical cell division and inhibited tissue reunion at the wound site.^46^ Moreover, grafted Arabidopsis GA biosynthesis and GA signaling mutants (*cps* and *cps rga gai* triple mutants, respectively) suppressed tissue reunion by inhibiting cortical cell expansion at the graft junction.^17^ These studies suggest that GA plays an essential role in the grafting process through cortical cell division and expansion at the graft junction. Therefore, GA accumulation at the incision site of the scion may play an essential role in the grafting process through cortical cell division and expansion.

### Jasmonic acid

#### Jasmonic acid and auxin accumulation showed a negative correlation at the incision site of the scion

**Figure 5.**
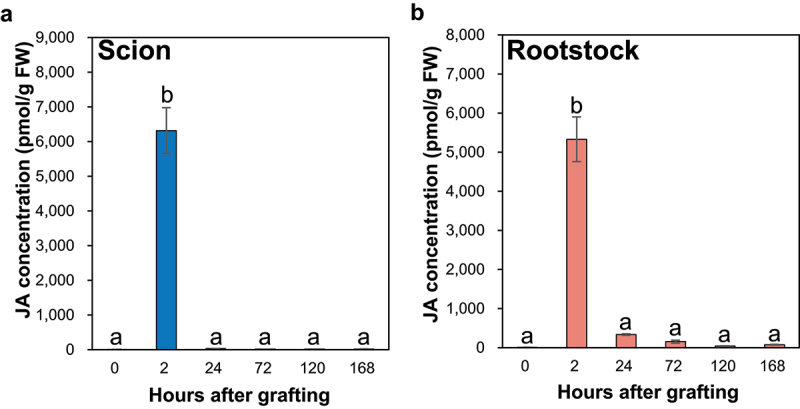
The JA concentration at the incision sites of the ungrafted scion and rootstock of *N.benthamiana*. Blue and pink indicate ungrafted scion and rootstock, respectively. Different letters indicate significant differences according to the Tukey-Kramer test (*p *≤ 0.05). Values are the means of four biological replicate samples, and error bars indicate the standard error of four biological replicate samples.

The JA concentration at the incision sites of the ungrafted scion and rootstock significantly increased at 2 HAC and increased approximately 790-fold at the incision site of the ungrafted scion (Figure 5(a)) and 670-fold at the incision site of the ungrafted rootstock compared with the control (Figure 5(b)). The JA concentration at the incision sites of the ungrafted scion and rootstock significantly decreased at 24 HAC compared to that at 2 HAC (Figure 5(a,b)). In addition, the JA concentration tended to be lower at 24 and 72 HAC (Figure 5(a,b)), whereas the IAA concentration was higher at 2 HAC at the incision site of the ungrafted scion than in the ungrafted rootstock (Figure 1(a,b)), leading to a negative correlation between the IAA and JA concentrations at the incision sites of the ungrafted scion and rootstock.

Auxins suppress JA biosynthesis. In incised Arabidopsis inflorescence stems, auxin transport from the apical bud to the root is blocked, and auxin levels increase in the upper part of the incised stem.^15^ Furthermore, in the upper part of the plant, auxin promotes the expression of *ARF6* and *ARF8*, suppressing the expression of the JA biosynthesis enzyme DEFECTIVE IN ANTHER DEHISCENCE 1 (DAD1).^15,16^ Consequently, JA levels decrease in the plant part above the incision compared to lower part.^15^

The negative correlation between JA and IAA concentrations at the incision sites of the ungrafted scion and rootstock in the present study suggests that at the incision site of the ungrafted scion, auxin levels increased owing to blocking of auxin transport from the apical bud to the root, auxin response factors ARF6 and ARF8 increased, and *DAD1* expression decreased, leading to lower levels of JA, whereas at the incision site of the ungrafted rootstock, auxin levels decreased and *DAD1* expression was not suppressed, leading to higher levels of JA. Jasmonic acid interacts with auxin and CK and regulates xylem formation by suppressing the expression of the auxin polar transporter PIN.^47^ Therefore, JA accumulation at the incision sites of the ungrafted scion and rootstock may play an important role in the grafting process through xylem formation.

#### Accumulation patterns of various plant hormones involved in cell division, callus formation, and xylem formation revealed at incision sites of the scion and rootstock

Plant hormones play essential roles in grafting.^14^ Based on the similarity between the tissue reunion processes of incised stems and grafting processes, Nanda and Melnyk^14^ suggested that plant hormones, such as auxin and JA, accumulate in graft junctions because of the expression of plant hormone biosynthesis and responsive genes in the parts above and below the incision. In the present study, we performed a comprehensive and long-term plant hormonome analysis of the incision sites of ungrafted scion and rootstock in *N. benthamiana* to determine which plant hormones accumulate at the graft junctions, whether these are supplied by the scion or rootstock, and their involvement in the grafting process. Consequently, different patterns of plant hormone accumulation were observed at the incision sites of the ungrafted scion and rootstock, as described below.

At the incision site of the ungrafted scion, JA levels were increased by stem incision (Figure 5(a)), and IAA levels were increased by blocking auxin transport from the apical bud to the root (Figure 1(a)). This increased IAA level may promote cell division in the pith tissue during the grafting process through auxin-responsive transcription factors, such as IAA1 and ANAC071. In addition, increased IAA levels may negatively regulate JA biosynthesis (Figures 1(a) and 5(a)). Furthermore, IAA inactivation from IAA to IAAsp by GH3 may suppress auxin-mediated CK biosynthesis, leading to an increase in the levels of iP-type CK at 72 HAC (Figures 1(a,c) and 2(c)). In contrast, at the incision site of the ungrafted rootstock, JA levels were increased by stem incision (Figure 5(b)), whereas IAA levels decreased because of the loss of auxin supply from the apical bud (Figure 1(b)). This reduction in IAA levels may not suppress CK biosynthesis by auxin, leading to an increase in the levels of tZ- and iP-type CKs from 24 HAC (Figures 1(b) and 2(b,d)). Abscisic acid accumulated at the incision sites of the ungrafted scion and rootstock and tended to accumulate more at the incision site of the ungrafted scion than of the ungrafted rootstock (Figure 3(a,b)), suggesting that ABA concentrations at the incision sites of the ungrafted rootstock and scion differed depending on the differences in ABA concentrations in the xylem and phloem sap.

These results indicate that plant hormones that regulate cell division, cell expansion, callus formation, and vascular formation regulate each other’s biosynthesis via crosstalk during the grafting process. This information is important for elucidating the molecular mechanisms of action of plant hormones during grafting.

#### Plant hormonome analysis of the intra- and inter-family graft junctions

Grafting is established through the following processes at the graft junction between the scion and rootstock: formation and degradation of the necrotic layer owing to collapsed cells, cell division and callus formation, intercellular junctions, plasmodesmata connection, and reformation and reconnection of vascular bundles.^11,12^ β-1,4-Glucanase promotes Arabidopsis self-grafting and inter-family grafting of *Nb/At* through necrotic layer degradation and cell wall reconstruction at the graft junctions,^12^ suggesting that the events from the early to the middle stages of the grafting process are similar between intra- and inter-family grafting. In contrast, partial tissue adhesion and no phloem connections were observed in the inter-family grafting of *Nb/At*, although plasmodesmata and xylem connections were observed, as in intra-family grafting,^48^ suggesting that the events from the middle to late stages of the grafting process, especially phloem formation and reconnection, are different between intra- and inter-family grafting.

**Figure 6.**
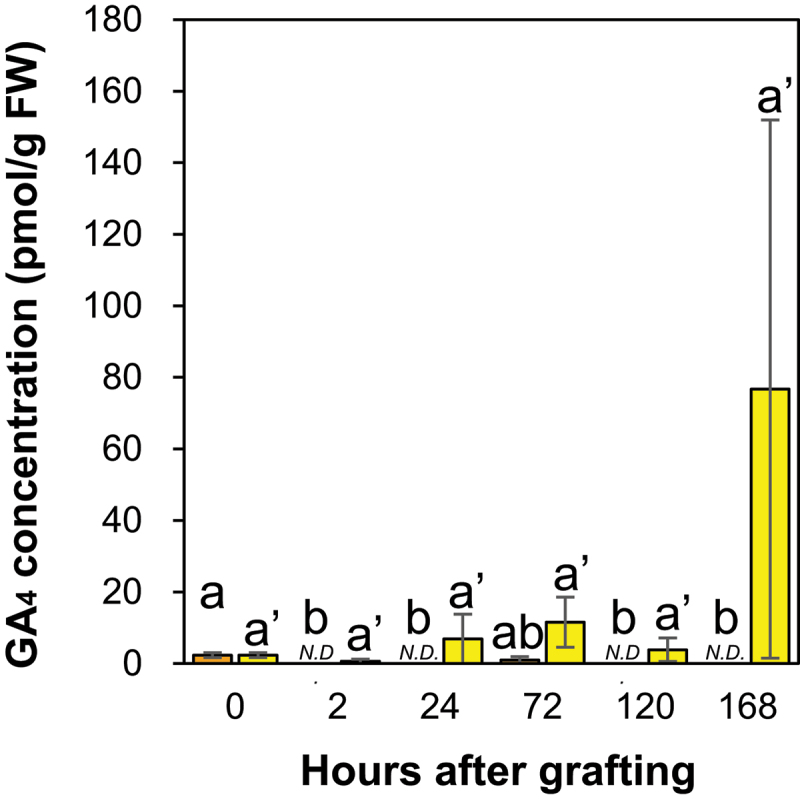
The GA_4_ concentration at the graft junctions of *Nb/Sl* and *Nb/At*. Orange and yellow indicate graft junctions of *Nb/Sl* and *Nb/At*, respectively. Different letters (a,b) and with dash (a’) indicate significant differences in each stage of the graft junctions of *Nb/Sl* and *Nb/At* according to the Tukey-Kramer test (*p* ≤ 0.05), respectively. Values are the means of four biological replicate samples, and error bars indicate the standard error of four biological replicate samples. *N.D*. indicates not detected.

In the present study, we performed a hormonome analysis of graft junctions of intra-family grafting (*Nb/Sl*: grafted plants with *N. benthamiana* as scion and *S. lycopersicum* as rootstock) and inter-family grafting (*Nb/At*: grafted plants with *N. benthamiana* as scion and *A. thaliana* as rootstock) to investigate the differences in the degree and speed of each event during the grafting process between intra- and inter-family grafting by determining the concentration and accumulation timing of plant hormones at the graft junctions. The levels of GA (GA_1_, GA_4_), ABA, SA, JA, auxin (IAA and IAAsp), and CKs (iP, tZ, cZ, and DZ types) were determined at the *Nb/Sl* and *Nb/At* graft junctions. GA_1_ was not detected, and GA_4_ was not significantly different between *Nb/Sl* and *Nb/At* graft junctions at each time point (Figure 6). In contrast, the concentrations of ABA, SA, JA, auxin, and CKs differed between *Nb/Sl*-and *Nb/At* graft junctions. The details and discussion are presented in the following sections.

### Auxins

#### Auxins accumulated in the graft junctions, and were highly accumulated at the graft junction of *Nb/At* compared with *Nb/Sl*

**Figure 7.**
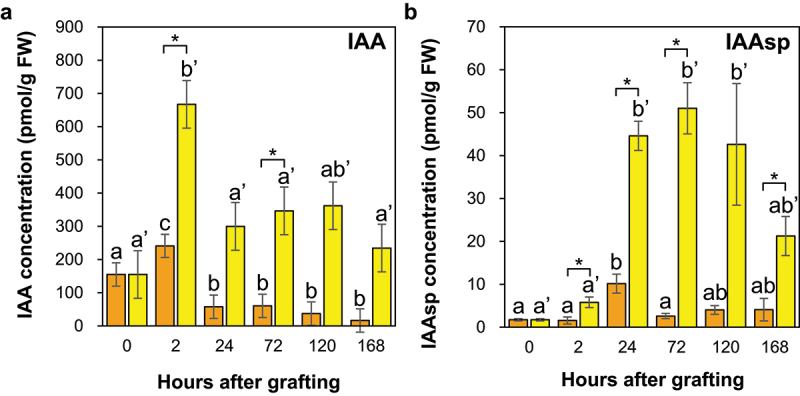
The auxin concentrations in the graft junctions of *Nb/Sl* and *Nb/At*. The concentrations of the IAA (a) and IAAsp (b) in the graft junctions of *Nb/Sl* and *Nb/At*. Orange and yellow indicate graft junctions of *Nb/Sl* and *Nb/At*, respectively. Different letters (a-c) and with dash (a’, b’) indicate significant differences in each stage of the graft junctions of *Nb/Sl* and *Nb/At* according to the Tukey-Kramer test (*p* ≤ 0.05), respectively. * indicates significant differences according to Student’s *t*-test (*p* ≤ 0.05). Values are the means of four biological replicate samples, and error bars indicate the standard error of four biological replicate samples.

The IAA concentrations at the *Nb/Sl* and *Nb/At* graft junctions significantly increased at 2 HAG and then significantly decreased compared to the control (Figure 7(a)). In incised Arabidopsis inflorescence stems, auxin transport from the apical bud to the root is blocked, and auxin levels increase in the part above the incision and decrease in the lower part.^15^ In the upper part, auxin promoted the expression of *ARF6* and *ARF8*, promoting the expression of *ANAC071*, and of *XTH19* and *XTH20*, which are downstream of *ANAC071*, and increased cell division of pith tissue during the tissue reunion process in incised inflorescence stems.^15,16^ Therefore, the increased IAA concentrations at the graft junctions of *Nb/Sl* and *Nb/At*, suggest that auxin response factors may promote both intra- and inter-grafting processes.

In addition, the IAA concentration at the graft junction was significantly higher at 2 and 72 HAG and tended to be higher at 2–168 HAG for *Nb/At* than for *Nb/Sl* (Figure 7(a)). A previous study reported that gene expression of the auxin-responsive transcription factors IAA1 and ANAC071 at the graft junctions at 1 DAG was higher in *Nb/At* than in self-grafted *N. benthamiana* plants.^12^ These results indicate that auxin accumulation and responses at the graft junctions were higher for inter-family grafting than for intra-family grafting. Auxin levels increased in the part above the incision by blocking auxin transport from the apical bud to the root. Thus, differences in auxin accumulation at the graft junction between *Nb/At* and *Nb/Sl* may reflect differences in the degree and speed of phloem recombination during the grafting process between intra- and inter-family grafting, or differences in IAA biosynthesis and transport activity between Arabidopsis and tomato used as scions.

#### Indole-3-acetic acid inactivation process by metabolizing to IAAsp promoted at the graft junction of *Nb/At* compared with *Nb/Sl*

The IAAsp concentration at the *Nb/Sl* graft junction significantly increased at 24 HAG and subsequently decreased compared to that in the control (Figure 7(b)). In contrast, the IAAsp concentration at the *Nb/At* graft junction significantly increased at 24, 72, and 120 HAG compared to the control (Figure 7(b)). The family of acyl acid amido synthetases GH3 catalyzes the conversion of IAA to IAA-amino acid conjugates such as IAAsp and indole-3-acetyl glutamate, and plays a central role in IAA inactivation.^37^ The IAA concentrations at the *Nb/Sl* and *Nb/At* graft junctions significantly increased at 2 HAG compared with the control and then significantly decreased (Figure 7(a)), suggesting that IAA accumulates at 2 HAG, whereas it is metabolized to IAAsp and inactivated by GH3 from 24 HAG at graft junctions of inter- and intra-family grafting.

In addition, the IAAsp concentration at the graft junction was significantly higher at 2, 24, 72, and 168 HAG, and tended to be higher at 120 HAG for *Nb/At* than for *Nb/Sl* (Figure 7(b)). The IAA concentration at the graft junction was significantly higher at 2 and 72 HAG and tended to be higher at 24–168 HAG for *Nb/At* than for *Nb/Sl* (Figure 7(a)). These results indicate that IAA accumulation and inactivation from IAA to IAAsp were higher in *Nb/At* than in *Nb/Sl* at the graft junction.

### Cytokinins

#### Cytokinin molecular types presented different accumulation patterns at the graft junctions of *Nb/Sl* and *Nb/At*

**Figure 8.**
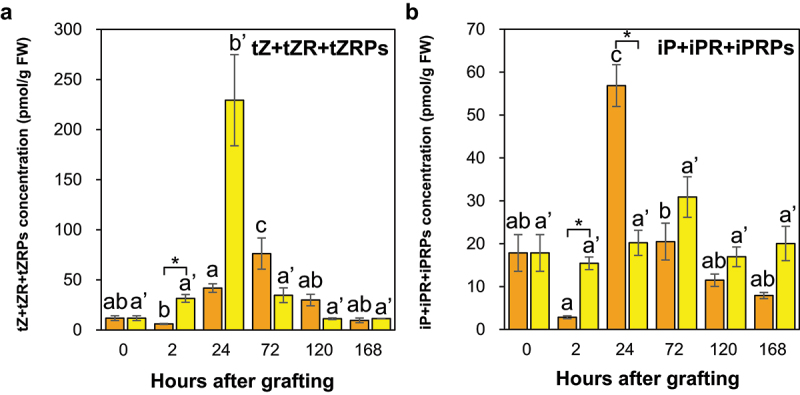
The major tZ- and iP-type CKs concentrations in the graft junctions of *Nb/Sl* and *Nb/At*. The total concentrations of the major tZ-type CKs (tZ, tZR, and tZRPs; a) and iP-type CKs (iP, iPR, and iPRPs; b) in the graft junctions of *Nb/Sl* and *Nb/At*. Orange and yellow indicate graft junctions of *Nb/Sl* and *Nb/At*, respectively. Different letters (a-c) and with dash (a’, b’) indicate significant differences in each stage of the graft junctions of *Nb/Sl* and *Nb/At* according to the Tukey-Kramer test (*p* ≤ 0.05), respectively. * indicates significant differences according to Student’s *t*-test (*p* ≤ 0.05). Values are the means of four biological replicate samples, and error bars indicate the standard error of four biological replicate samples.

The concentrations of the major tZ-type CKs (tZ, tZR, and tZRPs) tended to increase from 2 HAG and peaked at 72 HAG at the *Nb/Sl* graft junction, whereas they tended to increase from 2 HAG and peaked at 24 HAG at the *Nb/At* graft junction (Figure 8(a), Tables 3 and 4). In contrast, the concentrations of the major iP-type CKs (iP, iPR, and iPRPs) tended to increase from 2 HAG and peaked at 24 HAG at the *Nb/Sl* graft junction, whereas they tended to increase from 2 HAG and peaked at 72 HAG at the *Nb/At* graft junction (Figure 8(b), Tables 3 and 4). These results indicate that the iP- and tZ-type CK accumulation patterns differed between *Nb/Sl* and *Nb/At* graft junctions.

**Table 3.** The CK concentrations in the graft junctions of *Nb/Sl* grafting combination.

	Control	*Nb/Sl* _2 h	*Nb/Sl* _24 h	*Nb/Sl* _72 h	*Nb/Sl* _120 h	*Nb/Sl* _168 h
iP	*N.D*. a *N.D.*	a 0.25 ± 0.02 b*	*N.D*. a	*N.D. a*		*N.D*. a
iPR	0.28 ± 0.05 ab 0.06 ± 0.00	b* 1.03 ± 0.13 d*	0.72 ± 0.12 cd*	0.41 ± 0.04	bc*	0.49 ± 0.07 ac
iPRPs	17.57 ± 4.23 ab 2.78 ± 0.35	a* 55.59 ± 4.75 c*	19.77 ± 4.17 b	11.08 ± 1.39	ab	7.42 ± 0.66 ab
iP7G	3.17 ± 0.32 59.66 ± 5.17	* 79.07 ± 4.63 *	134.36 ± 6.22 *	**	-	** -
iP9G	*N.D*. a 0.01 ± 0.01	ab 0.01 ± 0.01 ab*	0.02 ± 0.02 ab	0.12 ± 0.05	b	0.08 ± 0.00 ab*
tZ	0.46 ± 0.09 ab 0.26 ± 0.01	b* 0.63 ± 0.05 ac*	0.77 ± 0.12 a*	0.31 ± 0.05	bc	0.19 ± 0.02 b
tZR	0.87 ± 0.12 a 0.25 ± 0.03	a* 4.12 ± 0.67 a	8.78 ± 1.80 b*	4.01 ± 0.83	a	1.64 ± 0.40 a
tZRPs	10.40 ± 2.15 ab 5.52 ± 0.50	b* 37.09 ± 3.57 a	66.73 ± 13.66 c	25.61 ± 4.89	ab	7.75 ± 1.89 ab
tZ7G	0.92 ± 0.16 a 1.47 ±	a 2.65 ± 0.22 a	13.87 ± 1.30 b*	19.97 ± 3.06	b	29.36 ± 3.45 c*
0.14 tZ9G	*N.D*. a *N.D.*	a* *N.D*. a*	*N.D*. a*	0.10 ± 0.05	b	0.07 ± 0.00 ab*
tZOG	0.06 ± 0.01 ab *N.D.*	b* 0.01 ± 0.01 b*	0.24 ± 0.06 ac*	0.23 ± 0.06	ac*	0.38 ± 0.06 c
tZROG	0.13 ± 0.03 a 0.06 ± 0.01	a 0.12 ± 0.01a*	1.10 ± 0.09 a	2.71 ± 0.25	b	3.60 ± 0.66 b
tZRPsOG	*N.D*. a *N.D.*	a *N.D*. a*	0.02 ± 0.02 a*	0.10 ± 0.01	b	*N.D.*
cZ	0.48 ± 0.37 a 0.10 ± 0.01	a* 0.07 ± 0.00 a*	0.02 ± 0.02 a*	0.02 ± 0.02	a	0.08 ± 0.01a*
cZR	0.10 ± 0.02 a 0.19 ± 0.03	ab* 0.21 ± 0.01 b	0.24 ± 0.01 b*	0.20 ± 0.02	b*	0.24 ± 0.02a
cZRPs	0.97 ± 0.12 a 1.23 ± 0.07	ab* 2.22 ± 0.05 c	1.68 ± 0.12 b*	1.69 ± 0.13	b	1.58 ± 0.12b*
cZOG	0.01 ± 0.01 a 0.04 ± 0.01	a *N.D*. a	0.02 ± 0.02 a	*N.D.*	a*	0.02 ± 0.02 ab*
cZROG	0.14 ± 0.03 a 0.04 ± 0.01	d 0.10 ± 0.01 ad*	0.19 ± 0.00 a*	0.29 ± 0.02	b	0.39 ± 0.02 c*
cZRPsOG	*N.D*. a *N.D.*	a *N.D*. a	*N.D*. a	*N.D.*	a*	*N.D*. a*
DZ	*N.D*. a *N.D.*	a 0.05 ± 0.00 a	0.04 ± 0.02 a	*N.D.*	a	0.02 ± 0.02 a
DZR	0.01 ± 0.01 a *N.D.*	a 0.07 ± 0.01 ac*	0.18 ± 0.02 b*	0.20 ± 0.02	b*	0.09 ± 0.02 c*
DZRPs	0.13 ± 0.03 a 0.11 ± 0.01	a 0.42 ± 0.06 ac*	0.77 ± 0.17 c	0.62 ± 0.07	bc*	0.24 ± 0.05 ab
DZ9G	0.08 ± 0.02 a *N.D.*	b *N.D*. b	*N.D*. b*	0.02 ± 0.01	b*	*N.D*. b*

The concentrations of iP, iPR, iPRs, iP7G, iP9G, tZ, tZR, tZRPs, tZ7G, tZ9G, tZOG, tZROG, cZ, cZR, cZOG, cZROG, DZ, DZR, DZRPs, and DZ9G. Different letters indicate significant differences in each stage of the graft junctions of *Nb/Sl* according to the Tukey-Kramer test (*p* ≤ 0.05), respectively. * indicates significant differences compared with *Nb/At* according to Student’s *t*-test (*p* ≤ 0.05), and ** indicates above the detection limit. The values represent the mean of four biological replicate samples. All concentrations are presented as means, and ± indicates the standard error of four biological replicate samples. *N.D*. indicates not detected.

**Table 4.** The CK concentrations in the graft junctions of *Nb/At* grafting combination.

	Control	*Nb/At* _2 h	*Nb/At* _24 h	*Nb/At* _72 h	*Nb/At* _120 h	*Nb/At* _168 h
iP	*N.D*. a	*N.D*. a	*N.D*. a*	0.11 ± 0.11 a	*N.D*. a	*N.D*. a
iPR	0.28 ± 0.05 a	0.33 ± 0.05 a*	0.34 ± 0.06 a*	2.39 ± 0.40 b*	1.31 ± 0.05 ab*	1.94 ± 0.46 b
iPRPs	17.57 ± 4.23 a	15.11 ± 1.43 a*	19.87 ± 2.88 a*	28.38 ± 4.24 a	15.63 ± 2.24 a	18.11 ± 3.56 a
iP7G	3.17 ± 0.32 a	7.12 ± 1.07 ad*	11.18 ± 1.08 ad*	53.88 ± 4.34 bd*	96.18 ± 22.59 b	151.66 ± 10.60 cd
iP9G	*N.D*. a	*N.D*. a	0.12 ± 0.02 a*	0.07 ± 0.01 a	0.36 ± 0.22 a	0.09 ± 0.04 a*
tZ	0.46 ± 0.09 a	0.91 ± 0.12 b*	1.24 ± 0.11 b*	0.29 ± 0.04 a*	0.17 ± 0.02 a	0.21 ± 0.02 a
tZR	0.87 ± 0.12 a	2.04 ± 0.22 b*	5.16 ± 0.34 c	1.15 ± 0.19 ab*	0.82 ± 0.05 a	1.26 ± 0.03 ab
tZRPs	10.40 ± 2.15 a	28.57 ± 3.61 a*	222.92 ± 45.20 b	33.21 ± 7.20 a	10.19 ±0.77 a	9.80 ± 0.19 a
tZ7G	0.92 ± 0.16 a	2.11 ± 0.25 a	3.43 ± 0.32 ac	7.38 ± 0.60 bc*	9.97 ± 2.07 b	9.31 ± 1.07 b*
tZ9G	*N.D*. a	0.08 ± 0.01 a*	0.37 ± 0.03 ac*	1.27 ± 0.13 bc*	1.76 ± 0.41 b	1.38 ± 0.19 b*
tZOG	0.06 ± 0.01 a	0.16 ± 0.02 ac*	0.39 ± 0.08 ab*	1.38 ± 0.22 b*	0.91 ± 0.18 ab*	1.21 ± 0.49 bc
tZROG	0.13 ± 0.03 a	0.27 ± 0.06 ad	0.78 ± 0.11 abd*	3.17 ± 0.56 c	2.40 ± 0.69 bc	2.18 ± 0.44 cd
tZRPsOG	*N.D*. a	*N.D*. a	0.22 ± 0.04 ab*	0.58 ± 0.09 b*	0.56 ± 0.17 b	0.48 ± 0.13 b*
cZ	0.48 ± 0.37 a	0.17 ± 0.03 a	0.16 ± 0.02 a*	0.16 ± 0.02 a*	0.22 ± 0.07 a	0.22 ± 0.04 a
cZR	0.10 ± 0.02 a	0.78 ± 0.08 d*	0.28 ± 0.04 ac	0.50 ± 0.02 bc*	0.48 ± 0.06 bc*	0.52 ± 0.03 b*
cZRPs	0.97 ± 0.12 a	6.32 ± 0.90 b*	5.39 ± 1.03 b	4.46 ± 0.33 bc*	4.36 ± 0.89 bc	3.74 ± 0.23 ab
cZOG	0.01 ± 0.01 a	*N.D*. a*	0.02 ± 0.02 a	*N.D*. a	0.17 ± 0.01 b*	0.14 ± 0.07 ab
cZROG	0.14 ± 0.03 a	0.17 ± 0.03 a	0.20 ± 0.01 ac*	0.34 ± 0.02 ab*	0.57 ± 0.16 b	0.50 ± 0.02 bc
cZRPsOG	*N.D*. a	*N.D*. a	0.02 ± 0.02 ac	0.07 ± 0.04 ab	0.19 ± 0.06 b*	0.15 ± 0.01 bc
DZ	*N.D*. a	*N.D*. a	0.14 ± 0.02 b	*N.D*. a	*N.D*. a	*N.D*. a
DZR	0.01 ± 0.01 a	*N.D*. a	0.13 ± 0.01 b*	0.09 ± 0.02 b*	*N.D*. a*	*N.D*. a*
DZRPs	0.13 ± 0.03 a	0.48 ± 0.09 a	3.86 ± 0.64 b*	1.31 ± 0.24 a	0.24 ± 0.04 a*	0.14 ± 0.02 a
DZ9G	0.08 ± 0.02 a	0.05 ± 0.03 a	0.05 ± 0.03 a	0.10 ± 0.03 a*	0.13 ± 0.02 a*	0.13 ± 0.02 a*

The concentrations of iP, iPR, iPRs, iP7G, iP9G, tZ, tZR, tZRPs, tZ7G, tZ9G, tZOG, tZROG, cZ, cZR, cZOG, cZROG, DZ, DZR, DZRPs, and DZ9G. Different letters indicate significant differences in each stage of the graft junctions of *Nb/At* according to the Tukey-Kramer test (*p* ≤ 0.05), respectively. * indicate significant differences compared with *Nb/Sl* according to Student’s *t*-test (*p* ≤ 0.05). The values represent the mean of four biological replicate samples. All concentrations are presented as means, and ± indicates the standard error of four biological replicate samples. *N.D*. indicates not detected.

The tZ-type CKs metabolize iP-type CKs via CYP735A.^49^ Therefore, these results suggest that CYP735A activity was high from 24 to 72 HAG at the *Nb/Sl* graft junction, which significantly increased the level of tZ-type CKs from 24 to 72 HAG, whereas CYP735A activity was low from 24 to 72 HAG at the *Nb/At* graft junction, which significantly decreased the level of tZ-type CKs from 24 to 72 HAG.

In addition, previous studies have suggested that tZ promotes shoot growth and wound-induced callus formation,^19,49^ whereas iP promotes polar auxin transport and vascular bundle formation in root meristematic tissues and regulates root development.^29^ In the present study, the molecular types of CKs at the *Nb/Sl* and *Nb/At* graft junctions showed different accumulation patterns (Figure 8; Tables 3 and 4). Therefore, these results suggest that the CK accumulation patterns might be related to the difference in the degree and speed of each event in the grafting process between intra- and inter-family grafting.

### Abscisic acid

#### Abscisic acid accumulated considerably at the graft junction of *Nb/At* compared with *Nb/Sl* at 24 HAG

**Figure 9.**
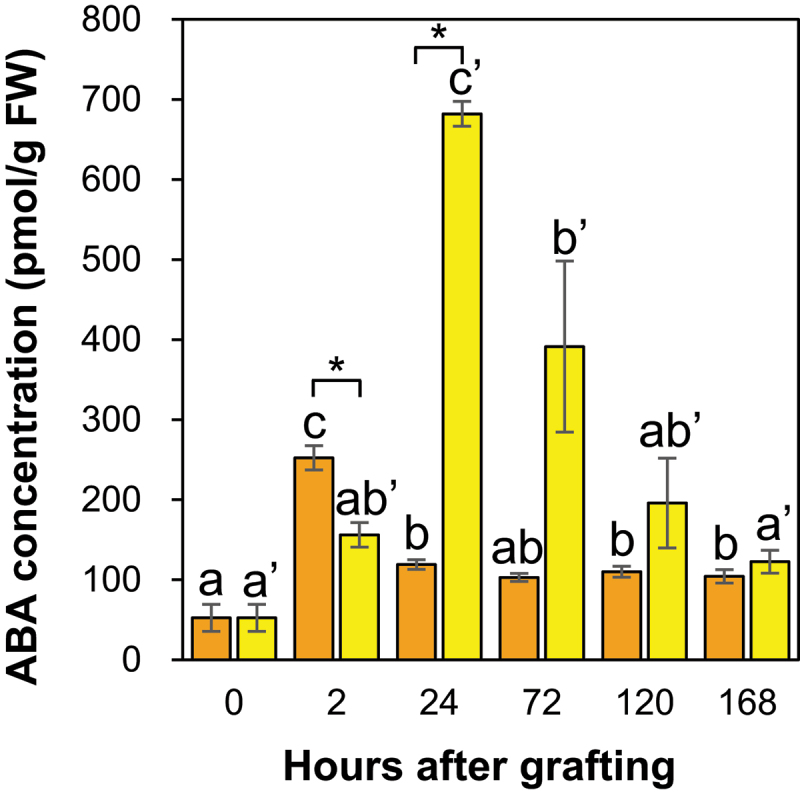
The ABA concentration in the graft junctions of *Nb/Sl* and *Nb/At*. Orange and yellow indicate graft junctions of *Nb/Sl* and *Nb/At*, respectively. Different letters (a-c) and with dash (a’-c’) indicate significant differences in each stage of the graft junctions of *Nb/Sl* and *Nb/At* according to the Tukey-Kramer test (*p* ≤ 0.05), respectively. * indicates significant differences according to Student’s *t*-test (*p* ≤ 0.05). Values are the means of four biological replicate samples, and error bars indicate the standard error of four biological replicate samples.

The ABA concentration at the *Nb/Sl* graft junction significantly increased by approximately 3-fold at 2 HAG and then significantly decreased compared to the control (Figure 9). In contrast, the ABA concentration in the *Nb/At* graft junction significantly increased by approximately 11-fold at 24 HAG and then significantly decreased compared to the control (Figure 9). A previous study suggested that ABA is primarily synthesized in leaf vascular cells, transported to the roots through the phloem, and then to the shoot through the xylem.^44^ Thus, ABA concentration significantly increased at the incision sites of *N. benthamiana* ungrafted scion and rootstock (Figure 3(a,b)), suggesting that ABA was transported through the phloem of the scion and xylem of the rootstock and accumulated at the *Nb/Sl* and *Nb/At* graft junctions.

In addition, the ABA concentration at the graft junction was significantly lower at 2 HAG for *Nb/At* than for *Nb/Sl* (Figure 9). These results may reflect differences in ABA supply through the xylem of *S. lycopersicum* and *A. thaliana* used as rootstocks. Furthermore, the ABA concentration at the graft junction was significantly higher at 24 HAG and tended to be higher at 72 and 120 HAG for *Nb/At* than for *Nb/Sl* (Figure 9). One reason for this difference in ABA accumulation may be the reduced water supply from the roots to the graft junctions through the xylem. The *N. benthamiana* scion experiences drought stress, which promotes ABA biosynthesis in the leaf vascular bundle cells and ABA transport to the graft junction via the phloem, which may result in a tended higher ABA concentration at the graft junction of *Nb/At* than of *Nb/Sl* from 24 HAG. Furthermore, in the inter-family grafting of *Nb/At*, although plasmodesmata and xylem connections were observed as in the intra-family grafting, partial tissue adhesion was observed, whereas phloem connections were absent.^48^ Therefore, at the graft junction of *Nb/Sl*, vascular bundle reconnection restored ABA transport between the scion and rootstock and eliminated ABA accumulation. In contrast, at the graft junction of *Nb/At*, the partial reconnection of the xylem and reduced water supply from the rootstock to the scion, resulting in drought stress to *N. benthamiana* as the scion, promoted ABA biosynthesis in the leaf vascular bundle cells and the transport of ABA to the graft junction via the phloem. In addition, the absence of phloem connections may result in higher ABA accumulation at the graft junction of *Nb/At* than of *Nb/Sl* from 24 HAG.

### Jasmonic acid

#### Jasmonic acid accumulation increases at the graft junctions, but differs between *Nb/Sl* and *Nb/At* graft junctions

**Figure 10.**
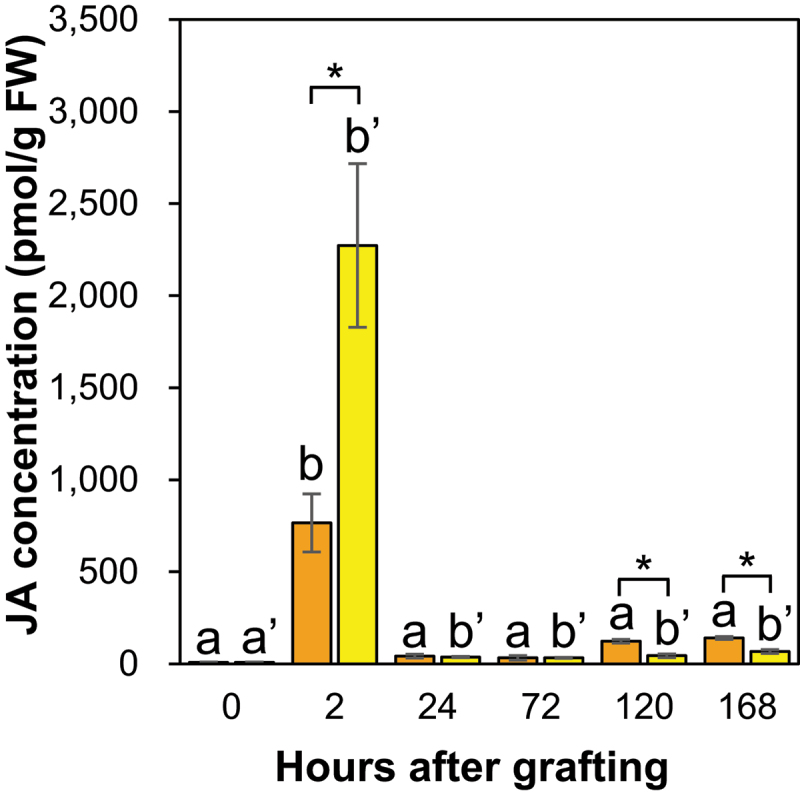
The JA concentration in the graft junctions of *Nb/Sl* and *Nb/At*. Orange and yellow indicate graft junctions of *Nb/Sl* and *Nb/At*, respectively. Different letters (a, b) and with dash (a’, b’) indicate significant differences in each stage of the graft junctions of *Nb/Sl* and *Nb/At* according to the Tukey-Kramer test (*p* ≤ 0.05), respectively. * indicates significant differences according to Student’s *t*-test (*p* ≤ 0.05). Values are the means of four biological replicate samples, and error bars indicate the standard error of four biological replicate samples.

The JA concentration at the graft junctions of *Nb/Sl* and *Nb/At* significantly increased at 2 HAG and decreased at 24 HAG compared to the control (Figure 10). The JA concentration at the graft junctions at 2 HAG was significantly higher for *Nb/At* than for *Nb/Sl* (Figure 10). *N.benthamiana* was used as the scion for the *Nb/Sl* and *Nb/At* grafted plants, suggesting that *S. lycopersicum* and *A. thaliana* were used as the rootstock to synthesize different amounts of JA, or *N. benthamiana*, used as the scion, recognized the rootstock plants and altered JA synthesis.

In addition, JA interacts with auxin and CK and regulates xylem formation by suppressing the expression of the auxin polar transporter PIN7.^47^ Therefore, this difference in JA accumulation may be related to the difference in the degree and speed of each event in the grafting process between intra- and inter-family grafting.

#### Jasmonic acid and auxin accumulation showed a negative correlation at the graft junctions of *Nb/Sl* and *Nb/At*

The JA concentration at the graft junction was significantly lower at 120 and 168 HAG for *Nb/At* than for *Nb/Sl* (Figure 10). In contrast, the IAA concentration at the graft junctions was significantly higher at 2 HAG and tended to be higher at 24 HAG for *Nb/At* than for *Nb/Sl* (Figure 8(a)). These results indicate a negative correlation between the IAA and JA concentrations at the graft junctions of *Nb/Sl* and *Nb/At*. In incised Arabidopsis inflorescence stems, auxin transport from the apical bud to the root is blocked, and auxin levels increase in the upper part of the incised stem.^15^ Furthermore, in the upper part of the incised stem, auxin promotes the expression of *ARF6* and *ARF8*, suppressing the expression of the JA synthase enzyme DAD1.^15,16^ As a result, JA levels decreased in the part above the incision compared with the lower part.^15^ Thus, a negative correlation was observed between IAA and JA concentrations at the graft junctions of *Nb/Sl* and *Nb/At* in the present study, suggesting that auxin suppressed JA biosynthesis at the graft junctions. Furthermore, the elevated IAA levels at the graft junction of *Nb/At* compared with *Nb/Sl* at 2 HAG (Figure 8(a)) suggested that auxin suppressed *DAD1* expression by promoting ARF6 and ARF8 expression, leading to lower JA levels at the graft junction for *Nb/At* than for *Nb/Sl* at 24 HAG (Figure 10).

### Salicylic acid

#### Salicylic acid and JA accumulation showed a negative correlation at the *Nb/Sl* and *Nb/At* graft junctions

**Figure 11.**
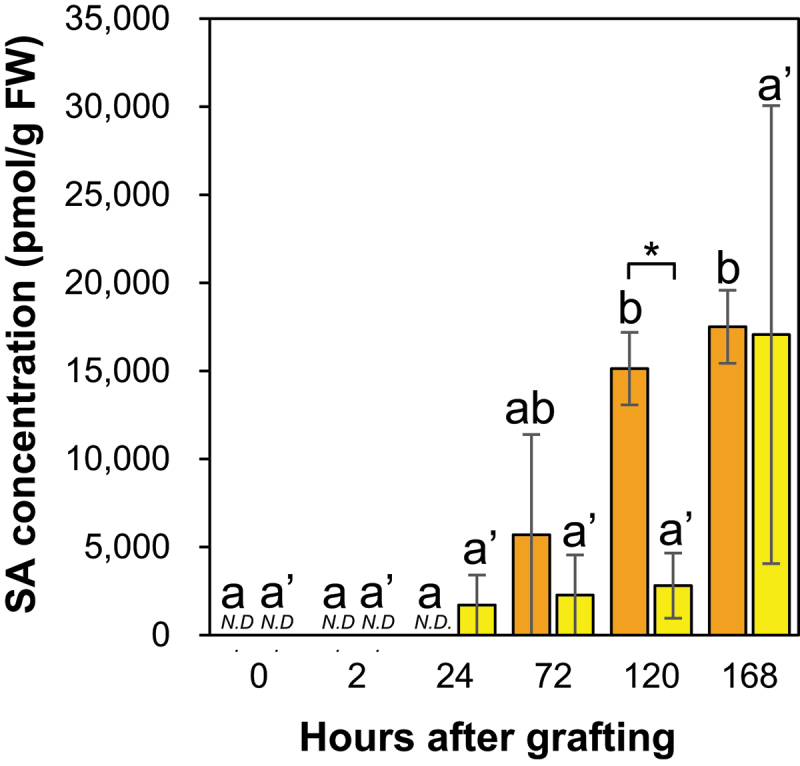
The SA concentration in the graft junctions of *Nb/Sl* and *Nb/At*. Orange and yellow indicate graft junctions of *Nb/Sl* and *Nb/At*, respectively. Different letters (a, b) and with dash (a’) indicate significant differences in each stage of the graft junctions of *Nb/Sl* and *Nb/At* according to the Tukey-Kramer test (*p* ≤ 0.05), respectively. * indicates significant differences according to Student’s *t*-test (*p* ≤ 0.05). Values are the means of four biological replicate samples, and error bars indicate the standard error of four biological replicate samples. *N.D*. indicates not detected.

Salicylic acid was not detected at 0, 2, and 24 HAG but was detected at 72 HAG, and the SA concentration increased over time at the *Nb/Sl* graft junction (Figure 11). In contrast, SA was not detected at 0 and 2 HAG, but was detected at 24 HAG at the *Nb/At* graft junction (Figure 11). In addition, the SA concentration was significantly lower at the graft junctions at 120 HAG for *Nb/At* than for *Nb/Sl* (Figure 11).

Salicylic acid interacts antagonistically with the JA biosynthetic pathway.^50,51^ Jasmonic acid concentration at the graft junctions of *Nb/Sl* and *Nb/At* significantly increased at 2 HAG and significantly decreased at 24 HAG, whereas the JA concentration at the graft junction at 2 HAG was significantly higher for *Nb/At* than for *Nb/Sl* (Figure 10). These results indicate a negative correlation between the SA and JA concentrations at the graft junctions of *Nb/Sl* and *Nb/At*, consistent with previous studies showing that SA interacts antagonistically with the JA biosynthetic pathway.^50,51^

#### Salicylic acid was detected at the graft junctions of *Nb/Sl* and *Nb/At*

Salicylic acid was not detected at the incision sites of the ungrafted scion and rootstock, whereas it was detected at the graft junctions of *Nb/Sl* and *Nb/At* from 72 HAG (Figure 11). These results indicate that SA is not synthesized and accumulates at the incision sites of the scion and rootstock with the incision of the inflorescence stems, whereas SA is synthesized and accumulated at graft junctions with grafting or accumulates in the stems of tomato and Arabidopsis used as rootstocks after grafting.

#### Accumulation patterns and concentrations of various plant hormones differed at the graft junctions of intra- and inter-family grafting

In this study, taking advantage of the ability of *N. benthamiana* to graft with many plant species, we performed a hormonome analysis of the graft junctions of *Nb/Sl* intra-family and *Nb/At* inter-family grafting to investigate the differences in the degree and speed of each event during the grafting process between intra- and inter-family grafting by determining the accumulation timing and concentration of plant hormones at the graft junctions.

Jasmonic acid and IAA levels were significantly increased at the *Nb/Sl* and *Nb/At* graft junctions at 2 HAG (Figures 7(a) and 10). The levels of IAA also significantly increased at the incision site of the ungrafted scion (Figure 1(a)), suggesting that the increases in IAA levels at the graft junction were caused by IAA accumulation at the incision site of the scion owing to the blocking of auxin transport from the apical bud to the root, and the promotion of the grafting process by auxin accumulation and auxin-responsive transcription factors in the intra- and inter-family grafts. The JA concentration in the graft junctions was significantly higher at 2 HAG for *Nb/At* than for *Nb/Sl* (Figure 10), suggesting that JA may strongly suppress SA biosynthesis in *Nb/At* compared to that in *Nb/Sl* graft junctions. The SA concentration at the graft junction from 24 HAG tended to be lower for *Nb/At* than for *Nb/Sl* (Figure 11). Furthermore, the IAA concentration at the graft junctions was significantly higher at 2 HAG and tended to be higher at 24 HAG for *Nb/At* than for *Nb/Sl* graft junctions (Figure 7A), whereas the JA concentration was significantly lower at 120 and 168 HAG for *Nb/At* than for *Nb/Sl* (Figure 10). These results suggest that IAA strongly suppresses JA biosynthesis at the graft junctions, leading to lower JA concentrations at 120 and 168 HAG for *Nb/At* than for *Nb/Sl*.

The ABA concentration at the graft junctions after 24 HAG was higher for *Nb/At* than for *Nb/Sl* (Figure 9). These results suggest that ABA accumulation at the *Nb/Sl* graft junction may be resolved by the reconnection of vascular bundles, whereas ABA accumulation at the *Nb/At* graft junction may not be resolved owing to the lack of phloem reconnection. The CK molecular types indicated different accumulation patterns at the graft junctions of *Nb/Sl* and *Nb/At* (Figure 8(a,b), Tables 3 and 4), suggesting that the CK accumulation patterns might be related to the difference in the degree and speed of each event in the grafting process between intra- and inter-family grafting.

This information is important for understanding the differences in molecular mechanisms between inter- and intra-family grafting and for elucidating the phenomenon of *N. benthamiana* compatible inter-family grafting with many plant species.

## Data Availability

All data are fully available without restriction.
